# Diet and Regimen, Physical, Intellectual, and Moral, as Means in the Prevention and Cure of Disease

**Published:** 1839-07

**Authors:** 


Art. VII.—*Diet and Regimen, Physical, Intellectual, and Moral, as Means in the Prevention and Cure of Disease.* By Robert Dick, m.d.—*Glasgow*, 1838. 8vo, pp. 386.

So many works on the subject which Dr. Dick has chosen for publishing a volume upon have appeared within the last few years, that it is difficult to conceive an author's inducement for appearing in the same course, unless he is conscious of possessing knowledge which has not hitherto been communicated to the public, or of powers of language more persuasive or convincing than that employed by his predecessors. Dr. Dick scarcely lays claim to either; and he sets out with what we pronounce to be a very erroneous notion, contained in the first sentence of his preface, that "It is *not* to be expected that on topics which are treated of in this volume, a work entirely original should be submitted to the public." It would be useless to dwell on Dr. Dick's frequent and somewhat curious appeals to his Glasgow townspeople; in which, at least, there is much originality. Perhaps he considered that they would read a book from him with more pleasure than one from an author unknown to them, and we hope he has found it so; but we must maintain that, as so many good and useful works are now to be found in every bookseller's shop, on the subject of Diet and Regimen, an author on these matters, if he cannot be original, would wisely keep his manuscript in his portfolio. Yet, probably, every work of this kind does good to somebody; and Dr. Dick's book is lively and pleasant enough, and may be read without any intellectual fatigue.

The portion of his work which he appears to consider peculiarly his own, that is to say, not taken from previous English or foreign and untranslated writers, nor even from Dr. Copland who has assisted Dr. Dick and seems to assist everybody with his multifarious knowledge, is that in which he treats of the mental and moral faculties, in three chapters: one on Our Intellectual Nature and its Regulation; one on Our Moral Nature and its Regulation; and one on "Love, Madness, Music;" which last might be a fragment of an unfinished novel. In the first of these chapters, the passages which have given us the most pleasure are those in which he recommends particular courses of reading for patients labouring under the influence of different passions. For the rest, many interesting subjects are touched upon; but the remarks are not such as to retain the attention or convey any novel instruction.

